# 686. Antibiotic Prescribing for Urinary Tract Infections in Outpatient Settings: A Multicenter Assessment Based on the Five Ds of Stewardship

**DOI:** 10.1093/ofid/ofaf695.225

**Published:** 2026-01-11

**Authors:** Amy Y Kang, Oliver Tan, Sunwoo Oh, Nikki Derleth, Megan Shieh, Helen Vu, Inna Tagarino, Audrey Harrington, Deborah Kupferwasser, Loren G Miller

**Affiliations:** Chapman University / Harbor-UCLA Medical Center, Irvine, CA; Chapman University School of Pharmacy, Irvine, California; Chapman University School of Pharmacy, Irvine, California; Chapman University School of Pharmacy, Irvine, California; Chapman University School of Pharmacy, Irvine, California; Chapman University School of Pharmacy, Irvine, California; Chapman University School of Pharmacy, Irvine, California; Chapman University School of Pharmacy, Irvine, California; Division of Infectious Diseases, the Lundquist Institute at Harbor-UCLA Medical Center, Torrance, CA, Torrance, California; Lundquist Institute at Harbor-UCLA Medical Center, Los Angeles, CA

## Abstract

**Background:**

Urinary tract infections (UTIs) are a leading cause of outpatient antibiotic use. Prior studies have documented inappropriate prescribing during outpatient UTI visits, but few have assessed intervention opportunities using a structured stewardship framework. We evaluated UTI prescribing practices in a healthcare system using the Five Ds framework: Diagnosis, Drug, Dose, Duration, and De-escalation.

**Table 1.**
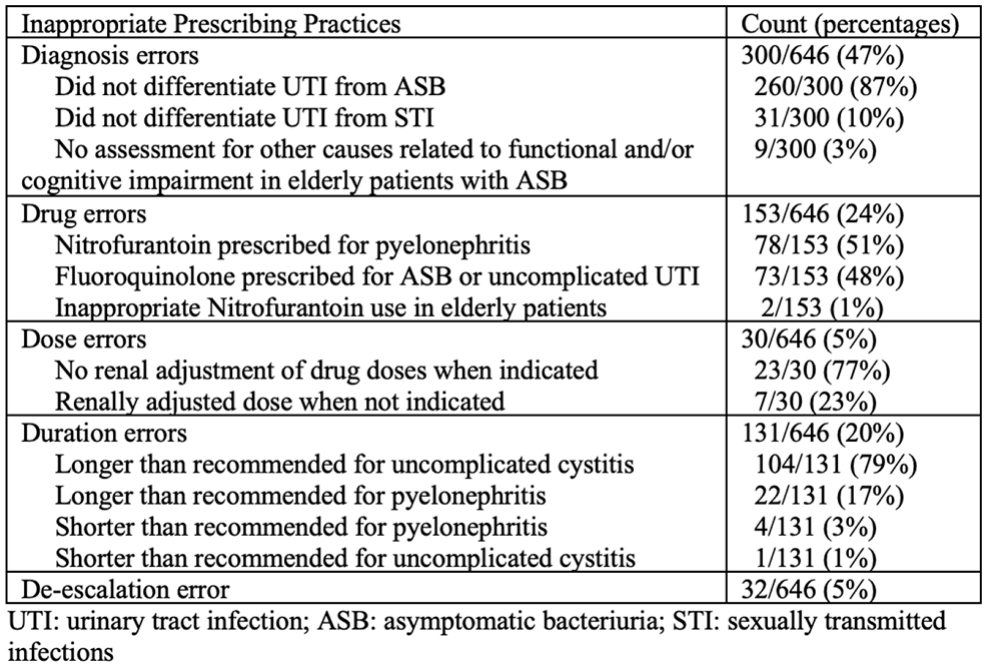
Breakdown of Inappropriate Prescribing Practices for Urinary Tract Infections (N=646) UTI: urinary tract infection; ASB: asymptomatic bacteriuria; STI: sexually transmitted infections

**Methods:**

We conducted a retrospective study across 4 medical centers and > 20 outpatient clinics within the Los Angeles County Department of Health Services, the second largest U.S. safety net healthcare system. To ensure adequate representation across sites while maintaining feasibility for manual chart review, we reviewed a random sample of adult patients with an ICD-10 code-based UTI diagnosis from 01/2019 to 01/2022. We excluded patients treated for non-UTI infections or not evaluated for acute UTI. For patients with multiple encounters, we included only the first episode. Prescribing practices were assessed for appropriateness using IDSA guideline criteria.

**Results:**

Of 48,149 UTI visits, we randomly selected and reviewed 2447 charts 1281 outpatient encounters met inclusion criteria for acute UTI. Of these, 646 (50%) had ≥ 1 inappropriate prescribing practice. Diagnostic errors were the most common (300/646, 46%), followed by drug selection (153/646, 24%), treatment duration (131/646, 20%), de-escalation (32/646, 5%), and dosing (30/464, 5%) (Table 1). Of the diagnosis errors, 260/300 (87%) were related to treatment of asymptomatic bacteriuria (ASB). Among drug-related errors, nitrofurantoin for pyelonephritis was most common (78/153, 51%), followed by fluoroquinolone use for ASB or uncomplicated UTI (73/153, 48%). The majority of duration-related errors were prolonged course for uncomplicated cystitis (104/131, 79%).

**Conclusion:**

In our health network, clinician-diagnosed UTI visits were commonly associated with incorrect diagnosis, inappropriate antibiotic selection, and inappropriately prolonged treatment. Our findings highlight opportunities for outpatient stewardship and targets for interventions to improve antibiotic prescribing.

**Disclosures:**

All Authors: No reported disclosures

